# Bilateral Adrenal Histoplasmosis in an Immunocompetent Host

**DOI:** 10.4269/ajtmh.21-0481

**Published:** 2021-08-23

**Authors:** Anthony Lieu, Deirdre Church, Stephen Vaughan

**Affiliations:** ^1^ Department of Medicine, Cummings School of Medicine, University of Calgary, Calgary, Alberta, Canada;; ^2^ Division of Infectious Diseases, Cummings School of Medicine, University of Calgary, Calgary, Alberta, Canada;; ^3^ Department of Pathology, Division of Microbiology, Cummings School of Medicine, University of Calgary, Calgary, Alberta, Canada

**Figure 1.**
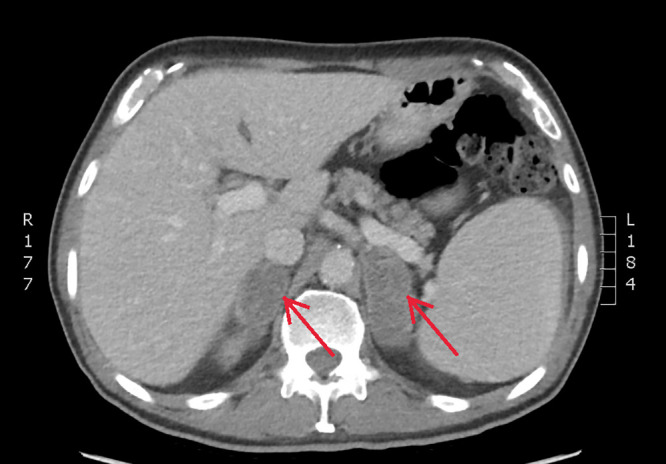
Computed tomographic (CT) scan of the abdomen, transverse view, showing bilateral adrenal masses (arrows) measuring 5.0 × 2.1 × 6.0 cm (anteroposterior [AP)], transverse [TV], craniocaudal [CC]) on the right and 6.1 × 2.6 × 5.7 cm (AP, TV, CC) on the left with heterogenous hypoattenuation. This figure appears in color at www.ajtmh.org.

**Figure 2.**
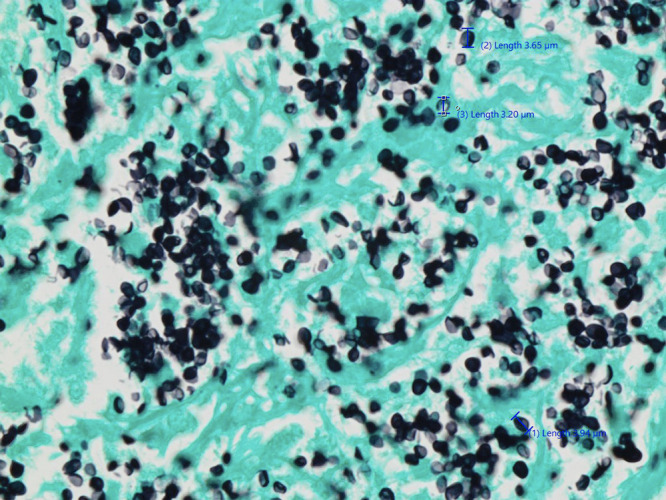
Findings from the adrenal tissue biopsy pathology with GMS stain, showing innumerable yeast measuring 2–4 µm. This figure appears in color at www.ajtmh.org.

**Figure 3.**
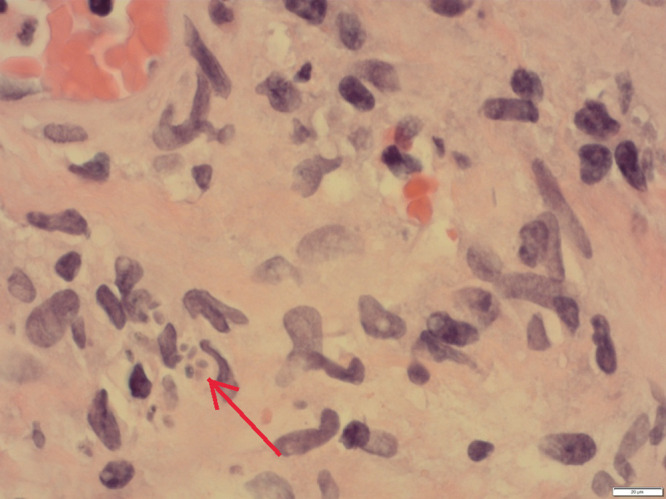
Findings from the adrenal tissue biopsy pathology with H&E stain, showing necrotizing granulomatous inflammation with purple pale staining clusters of round yeast-like organisms (arrow). This figure appears in color at www.ajtmh.org.

A 71-year-old healthy male presented with a 2-year history of intermittent fever, rigors, and night sweats. These symptoms were associated with loss of appetite and progressive weight loss of 50 lb. He was born in Canada and had traveled internationally, including extended periods in Germany, and more recently spending 6 months per year in the Philippines. No rash, lymphadenopathy or organomegaly was apparent. His laboratory results were remarkable for a sodium of 129 mmol/L and C-reactive protein (CRP) 27.7 mg/L, while his complete blood count was unremarkable. The cortisol level was normal but his adrenocorticotropic hormone was elevated at 17.8 pmol/L. Results of blood cultures, malaria real-time polymerase chain reaction (PCR) test, HIV, hepatitis B, and C serology were negative. A computed tomographic (CT) scan of his abdomen showed new bilateral adrenal masses with heterogenous hypoattenuation (Figure 1). He underwent a CT-guided biopsy. The diagnosis was established by histopathology with Grocott’s methenamine silver (GMS) stain of adrenal biopsy tissue sections, which showed small yeast (2–4 µm) with narrow-based budding (Figure 2). The H&E stain also showed purple pale staining clusters of round yeast-like organisms and extensive necrotizing granulomatous inflammation (Figure 3). Additional test results included a negative urinary Histoplasma antigen (Mayo Clinic, Rochester, MI), positive Histoplasma antibody immunodiffusion test (Alberta Provincial Laboratory for Public Health—North) and *Histoplasma capsulatum* growth on fungal culture of the adrenal tissue. An immunologic investigation only revealed an isolated low CD4 count of 210 cells/mm^3^, which increased on treatment to 397 cells/mm^3^ (normal range = 321–2,124 cells/mm^3^). He was treated with itraconazole at 200 mg PO TID for 3 days followed by itraconazole 200 mg PO BID with a planned duration of 1–2 years. He is currently 6 months into treatment with immediate resolution of fevers, rigors, and night sweats followed by improvement in fatigue, increased appetite, and 20 kg weight gain. To remediate his adrenal insufficiency, he was initiated on low-dose corticosteroids (hydrocortisone 10 mg PO qAM and hydrocortisone 5 mg PO qPM), with the guidance of an endocrinologist.

Histoplasmosis often presents with nonspecific symptoms including fever, night sweats, and progressive weight loss. The diagnosis is often delayed in a normal host, especially outside hyperendemic areas.^1^ Older adults without obvious immunosuppression may also develop chronic progressive disseminated histoplasmosis (CPDH), a slowly progressive disease with a high mortality.^2^ CPDH is thought to be caused by a specific defect in the immune response to this dimorphic fungi. Some experts suggest an immunodeficiency workup for this group of patients.^3^

Disseminated histoplasmosis commonly involves the adrenals, but adrenal insufficiency is quite rare as clinical adrenal insufficiency requires 90% destruction of the adrenal cortex.^4^ The differential diagnosis of infectious bilateral adrenal lesions is narrow and includes extrapulmonary tuberculosis, Waterhouse–Friderichsen syndrome from *Neisseria meningitis* infection, and dimorphic fungi (notably histoplasmosis and blastomycosis).^5^
